# Early Time-Restricted Eating Improves Weight Loss While Preserving Muscle: An 8-Week Trial in Young Women

**DOI:** 10.3390/nu17061022

**Published:** 2025-03-14

**Authors:** Zifu Yu, Takeshi Ueda

**Affiliations:** Graduate School of Humanities and Social Sciences, Hiroshima University, Higashi-Hiroshima 739-8524, Japan; d211386@hiroshima-u.ac.jp

**Keywords:** time-restricted eating, weight management, circadian rhythm, resistance training, nutritional timing

## Abstract

**Background**: Time-restricted eating (TRE) has gained attention as a novel dietary intervention that restricts the daily eating window, potentially offering improved metabolic health and body composition. Nevertheless, whether early TRE (eTRE) or delayed TRE (dTRE) best enhances resistance training (RT) adaptations remains unclear. **Methods**: In this 8-week randomized study, 24 healthy young women with limited RT experience were assigned into one of three groups: eTRE (an 8:00 AM–2:00 PM feeding window), dTRE (12:00 PM–6:00 PM), or the control (8:00 AM–8:00 PM). Apart from the timing restrictions, no further dietary guidance was provided. All of the participants performed standardized knee-supported push-ups (4 sets × 10 reps, three sessions/week). The primary outcomes included body weight, the thickness of the triceps brachii long head (measured via ultrasound), and push-up endurance. **Results**: The eTRE group achieved a significant reduction in body weight (−2.61 ± 1.06 kg; *p* < 0.001), which surpassed the changes observed in both the dTRE (−1.44 ± 1.12 kg) and control (−0.48 ± 0.64 kg) groups. However, no significant between-group differences emerged for muscle thickness or push-up performance. All groups showed comparable improvements in triceps brachii thickness (a 1.36–1.55 mm increase) and push-up endurance (62–74 additional repetitions). **Conclusions**: Early TRE (8:00 AM–2:00 PM) appears to be more beneficial than delayed TRE (12:00 PM–6:00 PM) for weight management when combined with RT, yet both TRE regimens result in similar improvements in muscle thickness and endurance. These findings suggest that optimizing meal timing in alignment with circadian rhythms may enhance weight control without hindering muscle adaptations, providing a practical approach for individuals seeking to lose weight while preserving or increasing their muscular fitness. Future research involving larger samples and diverse populations is warranted to confirm these results and clarify the underlying metabolic mechanisms.

## 1. Introduction

Skeletal muscle plays a pivotal role in maintaining human health and physical function, serving not only as the primary driving force for daily activities and various forms of exercise but also influencing one’s basal metabolic rate and overall energy balance [1,2]. With an aging population and increasingly sedentary lifestyles, sarcopenia has emerged as a pressing public health concern, often intertwined with obesity, diabetes, and cardiovascular diseases [3,4,5]. Consequently, identifying efficient strategies to preserve and enhance muscle mass has become a key focus in contemporary sports medicine and nutritional science.

Resistance training (RT) is well recognized as one of the most effective methods for increasing muscle strength and hypertrophy [6], and it also plays a crucial role in energy metabolism. Research has shown that it increases total energy expenditure both acutely, through exercise-induced energy consumption, and chronically, by elevating one’s resting metabolic rate due to muscle hypertrophy [2,6]. Additionally, resistance training contributes to improvements in body composition, reduces fat mass, and plays a significant role in weight management [7].

However, without proper dietary control, body weight and fat mass may rise in parallel with muscle gains. For individuals aiming to both gain muscle and reduce fat [8], appropriate dietary interventions are vital. Although traditional caloric restriction can help achieve weight control, it often leads to the loss of lean body mass and requires rigorous daily calorie calculations [9,10], resulting in relatively low adherence. This has prompted researchers and the public to seek novel dietary approaches that not only facilitate weight loss but also preserve or maintain lean mass better.

Time-restricted eating (TRE) is one such emerging dietary intervention that has recently garnered widespread attention. Its core principle is to confine the daily eating window to a relatively short duration—typically between 6 and 10 h—while remaining in a fasting or near-fasting state for the rest of the day [11,12]. Compared to conventional caloric restriction, TRE places greater emphasis on “when to eat” rather than “how much to eat” or “what to eat”, thereby reducing the need for precise calculations of food types or caloric content.

Existing research indicates that the metabolic benefits of TRE may be closely linked to circadian rhythms [13,14,15,16,17,18]. Many studies suggest that TRE not only helps to mitigate excessive energy intake but also helps to align meal timings with the body’s circadian rhythms. Additionally, by extending fasting periods, beneficial molecular changes have been reported, including enhanced fatty acid oxidation, improved insulin sensitivity, and the modulation of inflammatory and oxidative stress pathways [13,18,19,20,21,22,23]. The central biological clock, located in the suprachiasmatic nucleus (SCN) of the hypothalamus, primarily regulates the sleep–wake cycle and hormone secretion, whereas peripheral tissues, such as the liver, muscle, and adipose tissue, possess their own clock genes that respond acutely to feeding–fasting signals [14,24,25]. Both animal and human trials have demonstrated that TRE can effectively manage body fat and enhance metabolic health [12,26,27,28,29], while its relatively simple protocol fosters higher adherence. Notably, compared with conventional caloric restriction, TRE does not appear to significantly compromise the skeletal muscle during weight loss; in some cases, it may even facilitate the preservation of or a slight increase in lean mass [28,30,31,32,33].

Based on this, some researchers have begun to combine TRE with resistance training in an effort to optimize both muscle gain and fat reduction. Preliminary evidence suggests that across various training levels, TRE coupled with RT can reduce body fat while maintaining or increasing muscle mass. For instance, Moro et al. conducted a study on 34 healthy men with RT experience and found that in comparison to a normal-diet control group, an eight-hour TRE window (12:00 PM–8:00 PM) over eight weeks led to a more pronounced reduction in their body fat while maintaining similar gains in muscle mass and maximal strength [34]. Another eight-week randomized controlled trial involving young men with less RT experience used a four-hour eating window (selected within 12:00 PM–8:00 PM) for four days per week, alongside three RT sessions per week. Its results revealed that despite a reduction in their energy intake, the TRE group experienced no detrimental effects on lean body mass or muscle functionality; moreover, the improvements in both upper- and lower-limb strength, as well as lower-body muscular endurance, were more pronounced in the TRE group compared to these values in a standard three-meal control group [35]. However, it should be noted that most related studies have prescribed their eating windows around 12:00 PM–8:00 PM, likely reflecting modern schedules and social habits, in which dinner is a principal meal carrying social significance. Whether this noon-to-evening feeding window is the optimal approach remains unsubstantiated, and it overlooks the potential metabolic advantages of earlier feeding schedules.

In recent human studies, significant differences between “early TRE” and “delayed TRE” in terms of metabolic parameters such as fasting blood glucose and insulin sensitivity have been reported [36,37,38,39]. Similarly, in animal studies, delaying the TRE window can induce a phase shift in hepatic clock gene expression and disrupt normal lipid metabolism [40,41]. This may indicate that an earlier eating window aligns with the central and peripheral clocks better, thereby improving metabolic efficiency [11,42].

Resistance training is a key factor in promoting muscle growth or muscle hypertrophy, which is induced not only by mechanical stimuli but is also affected by metabolic byproducts such as lactate and circulating hormones [43]. Among these, anabolic hormones—including growth hormone (GH) and testosterone—play a crucial role in muscle adaptation. Notably, the secretion of these hormones follows circadian rhythms. For example, GH reaches its peak during the night, while testosterone levels are highest in the morning [44,45]. Additionally, insulin sensitivity exhibits diurnal variations, typically being higher in the morning [46], which may influence muscle nutrient utilization.

As an intervention strategy, time-restricted eating (TRE) may influence muscle adaptation by affecting hormone secretion patterns. This effect is partially mediated through the alignment of feeding times with circadian rhythms (e.g., early vs. delayed TRE). Consequently, different start times in TRE may exert varying impacts on muscle accretion and fat regulation, yet there remains a paucity of systematic research on this topic.

To explore the influence of early versus delayed TRE on body composition and metabolic health when combined with resistance training further, the present study aims to compare these two distinct TRE windows. Our findings will provide valuable evidence for developing individualized “muscle gain and fat loss” nutrition–exercise strategies. We hypothesize that early TRE, due to its stronger alignment with circadian rhythms, will lead to greater reductions in fat mass while preserving or even enhancing lean mass compared to delayed TRE.

## 2. Materials and Methods

### 2.1. Ethical Approval and Participants

This study was approved by the Ethics Committee of Hiroshima University (Approval No. HR-ES-001701, approved on 21 May 2024) and was conducted in accordance with the principles outlined in the Declaration of Helsinki. All participants provided their written informed consent after being informed of this study’s objectives, procedures, potential risks, and expected benefits.

Participants were recruited from 10 June 2024 to 1 July 2024. To minimize sex-related metabolic variability, only healthy female university students (aged 18–26 years) were recruited via campus social media platforms. The inclusion criteria were as follows:

(1) No regular resistance training experience in the previous six months;
(2) No serious musculoskeletal or cardiovascular disorders;
(3) Non-smokers and non-heavy drinkers.

A total of 29 volunteers initially met these criteria and subsequently underwent a one-week familiarization period to help them adapt to the time-restricted feeding schedule and become familiar with the push-up technique. During this period, three volunteers withdrew due to difficulties adapting to resistance training, and two withdrew due to fasting-related discomfort. Consequently, 24 participants proceeded to the formal eight-week intervention.

The participants were randomly allocated, via computer-generated sequences and sealed envelopes, into three groups: an early time-restricted eating group (eTRE), a delayed time-restricted eating group (dTRE), and a control group.

### 2.2. Interventions

This study did not alter the participants’ usual diet composition or caloric intake but rather adjusted the duration and timing of their daily feeding windows as follows:

- **The eTRE group** (early time-restricted eating): 8:00 AM–2:00 PM, permitting ±30 min flexibility for the first and last meal;
- **The dTRE group** (delayed time-restricted eating): 12:00 PM–6:00 PM, also permitting ±30 min flexibility in their meal timing;
- **The control group**: 8:00 AM–8:00 PM, with no additional time restrictions.

Daily phone calls or online messages were used to monitor the groups’ compliance with each designated feeding window, as well as to record the participants’ sleep durations and quality. If the participants had to adjust their meal times due to social events or their academic schedules, these deviations were recorded, and their impact on the outcomes was accounted for in the final statistical analysis.

To simulate a real-life TRE implementation better, no strict caloric restrictions were imposed. However, the participants were required to consume at least 1.2 g/kg body weight of protein daily to minimize potential confounding effects on their muscle adaptation. Compliance with protein intake was monitored through three-day dietary photo logs and subjective dietary descriptions. Apart from these dietary considerations and meal timing restrictions, no further dietary guidance or limitations were implemented.

All of the participants also performed a standardized push-up protocol over an eight-week period. The exercise involved a knee-supported push-up to reduce the load while maintaining the proper technique (i.e., aligning the torso and thighs, lowering the body until the elbows reach approximately 90° flexion, and then pushing back up to near-full extension). The rhythm was two seconds down and one second up for each repetition, and a metronome was used to ensure a consistent pace throughout the training sessions and endurance assessments. Each training session comprised four sets of ten repetitions, with 90 s of rest between sets, performed three times per week (e.g., Monday, Wednesday, and Friday). Based on prior research indicating that when the total training volume is comparable, muscle adaptations remain relatively consistent despite differences in the load intensity [47], we used fixed sets and repetitions to minimize inter-individual variability. Investigators continuously supervised each training session to correct the participants’ form and record the actual number of repetitions completed per set. If a participant was unable to complete all ten repetitions in a set, the successful repetitions were noted; participants were encouraged to aim for ten repetitions in subsequent sessions. All training sessions were logged to evaluate exercise adherence.

### 2.3. Outcome Measurements

Assessments were conducted at baseline (Week 0) and after eight weeks of intervention (Week 8). The measurements took place in a sports physiology laboratory, generally in the morning under fasted conditions to ensure consistent testing circumstances.

- **Body weight**: This was measured using a calibrated digital scale with 0.1 kg precision. The participants wore light clothing and were instructed to empty their bladder prior to measurement.
- **Thickness of the long head of the triceps brachii**: This was assessed using an ultrasound device (LV8-4L65S-3, Telemed, Vilnius, Lithuania). The participants were seated, with their forearms resting on a table with their palms facing downward. The midpoint between the acromion and the olecranon was marked, and a water-soluble ultrasound gel was evenly applied. The transducer was placed vertically to measure muscle thickness. Each participant underwent three measurements, and the mean value was used for analysis. All measurements were performed by the same experienced technician to minimize inter-rater variability. The triceps brachii was selected as the primary muscle for analysis for two main reasons. First, push-ups effectively stimulate the triceps brachii, making it a relevant muscle to assess in this study. Second, compared to other muscle groups, the triceps brachii is more accessible for measurement, allowing for greater accuracy and reproducibility in ultrasound assessments.
- **Muscular endurance**: This was defined as the maximum number of knee-supported push-ups the participant could complete in a single test session, summing the repetitions from up to four sets. The first test was performed at baseline, and the second was scheduled at least 48 h after the final training session to minimize the effects of fatigue. Participants were instructed to maintain proper form; once they could no longer meet the standard, testing stopped, and the last valid repetition was recorded.

### 2.4. Statistical Analysis

All statistical analyses were conducted using SPSS (version 27.0, IBM Corporation, Armonk, NY, USA). The significance level was set at *p* < 0.05, and all results were reported as the mean ± standard deviation (mean ± SD).

Due to the relatively small sample size (n = 8 per group), a linear mixed model (LMM) was employed instead of a traditional repeated-measures ANOVA to enhance the statistical power and account for individual variability. This approach allowed for a robust estimation of the within-subject and between-group effects.

The dependent variables were body weight (kg), muscle thickness (mm), and push-up repetitions, with Time (pre- vs. post-intervention) and Group (eTRE, dTRE, and control) as fixed effects, along with a term for their interaction (Time × Group). In the LMM, the Group variable was dummy-coded, with the control group designated as the reference category. Participant IDs were included as random intercepts to account for individual differences.

The primary focus of the analysis was the Time × Group interaction, which determined whether the changes in the dependent variables differed across groups. If a significant interaction was detected (*p* < 0.05), post hoc pairwise comparisons were performed using Tukey’s honestly significant difference (HSD) test to identify specific group differences while adjusting for multiple comparisons. All of the models were fitted using restricted maximum likelihood estimation (REML).

## 3. Results

### 3.1. Physical Characteristics

The participants’ physical characteristics are presented in Table 1, while Table 2 presents the pre- to post-intervention changes and the results of the analysis using the linear mixed model (LMM).

### 3.2. Adherence to Eating Time and Sleep Duration

Table 3 presents the rates of adherence to the designated eating times and sleep durations across all groups during the study period. The participants exhibited high adherence rates to the time-restricted eating schedules (eTRE group, 85.6%; dTRE group, 89.5%), indicating that these dietary patterns are highly feasible and acceptable in real-life settings. Additionally, there was no significant difference in sleep duration between the time-restricted eating groups, with the eTRE, dTRE, and control groups averaging 7.66 ± 0.52, 7.49 ± 0.48, and 7.58 ± 0.45 h, respectively.

### 3.3. Changes in Body Weight

**Figure 1:**
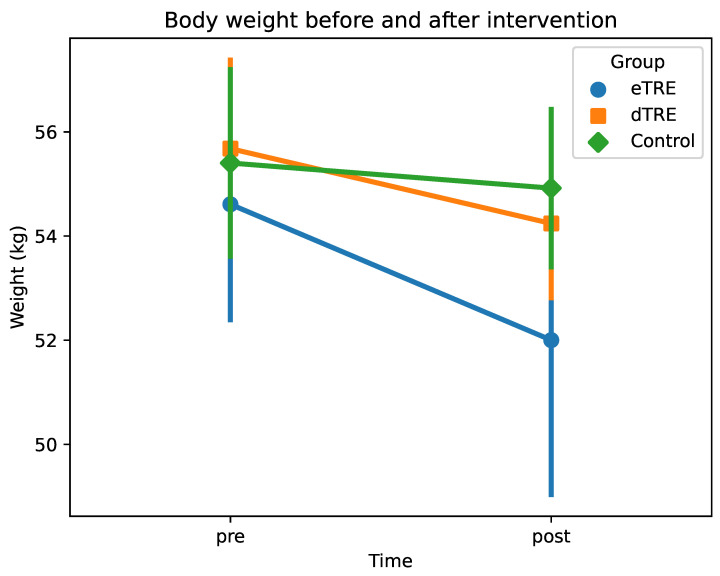
Body weight (kg) before and after intervention. Mean body weight (kg) at two time points (pre vs. post) for each group (eTRE, dTRE, and control). Markers (circles, squares, and diamonds) represent group means, and error bars indicate ±1 standard deviation (SD).

After the intervention, eTRE had a lower weight than the control group (β = −2.92; SE = 1.00; 95% CI: [−4.88, −0.95]; *p* = 0.004), whereas dTRE did not differ significantly from the control (β = −0.68; SE = 1.00; 95% CI: [−2.64, 1.29]; *p* = 0.499). The Time × Group interaction term for eTRE (β = 2.13; SE = 0.48; 95% CI: [1.18, 3.07]; *p* < 0.001) indicated that eTRE experienced a significantly greater reduction in weight from pre to post compared to the control. In contrast, the interaction term for dTRE (β = 0.96; SE = 0.48; 95% CI: [0.01, 1.90]; *p* = 0.047) suggested a moderate weight reduction, though a smaller reduction than that observed in eTRE. Tukey’s HSD post hoc tests showed that the post-intervention weight in eTRE was significantly lower than that in the pre-control (*p* = 0.018) and pre-dTRE (*p* = 0.008) groups, but the difference between the post-eTRE and post-control groups did not reach statistical significance (*p* = 0.060). Taken together, these findings suggest that eTRE led to the most pronounced weight reduction, whereas dTRE and the control experienced smaller and more comparable changes. The changes in body weight before and after the intervention are shown in Figure 1.

### 3.4. Changes in the Thickness of the Long Head of the Triceps Brachii

After the intervention, eTRE showed a greater muscle thickness compared to that in the control (β = 1.53; SE = 0.75; 95% CI: [0.05, 3.00]; *p* = 0.043), whereas dTRE did not differ significantly from the control (β = 0.91; SE = 0.75; 95% CI: [−0.57, 2.38]; *p* = 0.228).

The interaction terms for Time × Group were not significant for either eTRE (β = 0.08; SE = 0.28; 95% CI: [−0.47, 0.63]; *p* = 0.780) or dTRE (β = 0.19; SE = 0.28; 95% CI: [−0.37, 0.74]; *p* = 0.506), suggesting that the pre–post changes in muscle thickness followed a similar trend across groups.

**Figure 2:**
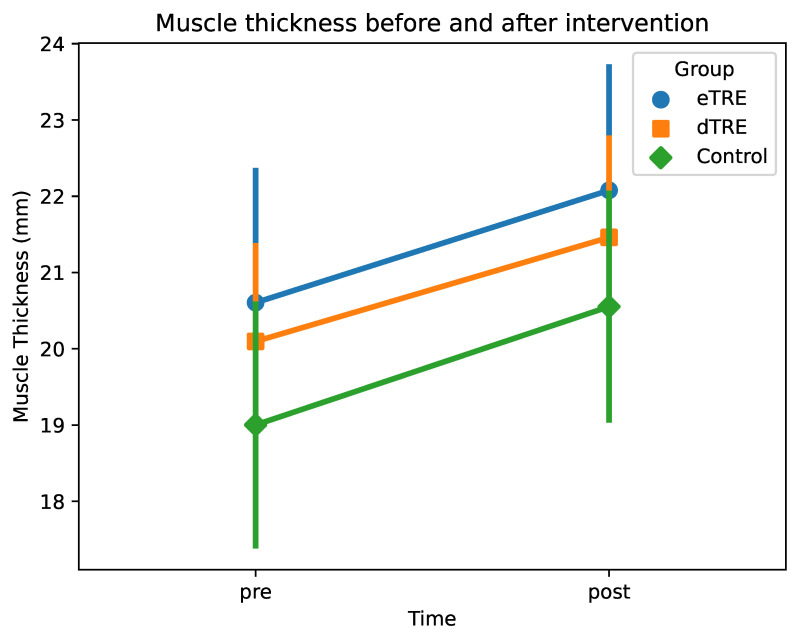
Muscle thickness (mm) before and after intervention. Mean muscle thickness (mm) at two time points (pre vs. post) for each group (eTRE, dTRE, and control). Markers (circles, squares, and diamonds) represent group means, and error bars indicate ±1 standard deviation (SD).

Taken together, these findings suggest that eTRE led to a greater muscle thickness compared to that in the control after the intervention. However, the pre–post changes in muscle thickness followed a similar pattern across groups. The changes in muscle thickness (mm) before and after the intervention are shown in Figure 2.

### 3.5. Changes in Push-Up Performance

Neither dTRE (β = −11.88; SE = 12.42; 95% CI: [−36.23, 12.48]; *p* = 0.339) nor eTRE (β = −1.63; SE = 12.42; 95% CI: [−25.98, 22.73]; *p* = 0.896) showed significant differences in their push-up repetitions post-intervention. The wide confidence intervals suggest high variability in the individual responses.

Similarly, the interaction terms for Time × Group were not significant for either dTRE (β = 6.00; SE = 8.62; 95% CI: [−10.89, 22.89]; *p* = 0.486) or eTRE (β = −6.00; SE = 8.62; 95% CI: [−22.89, 10.89]; *p* = 0.486). This suggests that while slight differences in the pre–post changes were observed between groups, these differences were not statistically meaningful.

Tukey’s HSD post hoc tests indicated that the within-group comparisons showed significant improvements in the push-up repetitions from pre to post across all groups (*p* < 0.001). However, no significant differences were found between groups.

**Figure 3:**
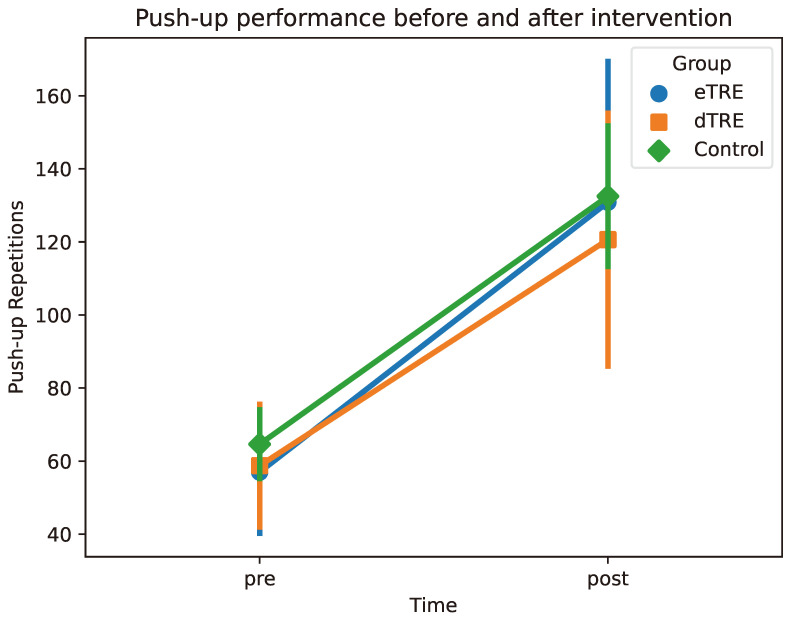
Push-up performance before and after intervention. Mean push-up repetitions at two time points (pre vs. post) for each group (eTRE, dTRE, and control). Markers (circles, squares, and diamonds) represent group means, and error bars indicate ±1 standard deviation (SD).

Taken together, these findings suggest that the push-up endurance improved significantly over time in all groups, but the degree of improvement was comparable among the eTRE, dTRE, and control groups. The changes in the push-up repetitions before and after the intervention are shown in Figure 3.

## 4. Discussion

This study investigated how two distinct time-restricted eating (TRE) patterns—early TRE (eTRE) and delayed TRE (dTRE)—affect body weight, muscle hypertrophy, and muscle endurance in young women undergoing resistance training. The findings indicate that eTRE outperforms dTRE in weight management. However, despite the differences in the meal timing, all groups demonstrated comparable improvements in their muscle thickness and endurance. In the following sections, we delve into these primary results and their possible mechanisms in light of previous research while also discussing secondary findings related to sleep quality and adherence, as well as this study’s limitations and the prospects for future research.

### 4.1. Significant Weight Loss Effects of eTRE

A principal outcome of this study is that eTRE exhibited a marked advantage in terms of weight management, whereas dTRE showed a moderate weight reduction that approached statistical significance (*p* = 0.047). Compared with the control group, the average body weight in the eTRE group decreased by 2.61 ± 1.06 kg, a statistically significant reduction (β = 2.13, *p* < 0.001). In contrast, the dTRE group showed an average decrease of 1.44 ± 1.12 kg, which approached statistical significance (β = 0.96, *p* = 0.047) but had a smaller effect size than that for eTRE. This result aligns with certain previous findings. For instance, in a five-week randomized controlled trial involving non-obese, healthy participants, Xie et al. reported that eTRE—compared with dTRE—produced more pronounced benefits in improving insulin sensitivity and fasting glucose levels, in addition to reducing overall body weight and adiposity [37]. Animal models also suggest that eTRE is more closely aligned with the body’s circadian rhythm genes. In an eight-week randomized controlled trial in mice, eTRE not only reduced body weight more significantly than dTRE but also helped maintain synchronization of the liver’s circadian clock [41]. Moreover, Richter et al. found that the thermic effect of food (TEF)—the energy expenditure required for digestion, absorption, and the metabolism of nutrients—after breakfast is 2.5 times higher than that after dinner [48], further explaining how eTRE could achieve significant weight loss by enhancing metabolic efficiency.

However, the dTRE results herein differ from the findings of some earlier studies. Moro et al., for example, reported that in men undergoing resistance training, an eight-week dTRE protocol (12:00 PM–8:00 PM) significantly reduced their fat mass and body weight compared with these values in a control group (regular three daily meals), even under identical energy intake conditions [34]. Moreover, a systematic review by Adafer et al. suggested that both eTRE and dTRE can significantly reduce total energy intake—by about 20%—and lead to an approximate 3% decrease in body weight [21]. The non-significant weight loss from dTRE in this study may be attributed to the participants’ actual energy intake, lower metabolic efficiency in the evening, and differences in the experimental design. For instance, dinner is often considered the most substantial meal in many cultures, meaning the individuals in the dTRE group may have consumed greater overall calories or more energy-dense foods in the evening, offsetting the energy deficit TRE might have otherwise imposed. Additionally, some studies indicate that delayed eating can raise the risk of nighttime fat storage and lower total energy expenditure [49,50]. Furthermore, this study did not record the participants’ energy intake and macronutrient distribution in detail, potentially masking any weight loss benefits of dTRE.

In summary, the significant weight loss effect of eTRE may be linked to its closer alignment with the human circadian rhythm, whereas the impact of dTRE may be constrained by the reduced metabolic efficiency resulting from delayed meal timing.

### 4.2. The Limited Influence of TRE on Muscle Hypertrophy and Endurance

Despite the pronounced weight reduction observed in the eTRE group, no significant differences were found among the groups in terms of muscle thickness and endurance. This suggests that TRE may not greatly affect resistance-training-induced muscle adaptations. These findings are consistent with those of Moro et al., who examined an eight-week dTRE protocol (12:00 PM–8:00 PM) combined with resistance training in trained, healthy men. Although their levels of testosterone and insulin-like growth factor-1 (IGF-1) decreased significantly, their fat-free mass and the cross-sectional area of their limb muscles were comparable to those in the group following a conventional meal schedule [34]. Similarly, Tinsley et al. reported that in young men undergoing resistance training, the dTRE group—despite consuming about 650 fewer kilocalories on average—did not differ significantly from a control group in terms of the gains in their leg strength and muscle endurance [35]. Another study on active women showed that with a similar energy and protein intake, dTRE does not diminish skeletal muscle hypertrophy or performance after resistance training [30]. This study is the first to incorporate eTRE with resistance training, and we similarly observed no distinct effects on muscle adaptation compared with dTRE.

Several factors may have contributed to the limited effect of TRE on muscle growth. First, the rate of myofibrillar protein synthesis (MyoPS) directly influences muscle protein accumulation [51]. A recent study in overweight/obese males indicated that an eight-hour TRE window (10:00 AM–6:00 PM) does not reduce daily MyoPS rates relative to those under a twelve-hour, isocaloric eating regimen [52]. Second, TRE exerts bidirectional hormonal effects that may balance each other out in terms of muscle adaptation. On the one hand, TRE enhances insulin sensitivity and significantly increases the expression of the primary nutrient-sensing protein mammalian target of rapamycin (mTOR), which regulates cell growth [19]. On the other hand, it may lower testosterone and IGF-1 levels, both of which are crucial for protein synthesis and muscle repair [53,54]. Such competing hormonal responses may explain why eTRE and dTRE showed no significant differences in muscle adaptations.

Finally, the absence of differences between eTRE and dTRE may have been due to resistance training’s dominant role in muscle adaptation. Compared with weight management, muscle development may be less sensitive to changes in meal timing. Additionally, the short intervention period or a lack of variety in the training stimuli could have concealed potential discrepancies. Resistance training is the primary stimulus for muscle hypertrophy, as it induces mechanical tension and metabolic stress, which in turn trigger muscle protein synthesis (MPS) [6]. In addition to these stimuli, the frequency, intensity, and volume of resistance training play a crucial role in optimizing muscle growth and strength adaptation.

Adequate nutrient intake is another key factor in supporting muscle repair and development, with protein consumption being particularly important. A daily protein intake of 1.2–2 g/kg body weight is generally recommended to maximize MPS. Additionally, energy intake is essential, as a caloric deficit may impair MPS and limit hypertrophy, whereas sufficient energy availability supports lean mass gains when combined with resistance training.

Furthermore, adequate sleep and recovery are vital for muscle repair and adaptation. Poor sleep quality or insufficient recovery can disrupt anabolic hormone secretion, negatively affecting muscle growth. For instance, growth hormone (GH) secretion peaks during sleep, playing a crucial role in muscle preservation and fat metabolism. Individuals following time-restricted eating (TRE) should ensure sufficient sleep to maximize the benefits of muscle adaptation and overall recovery. Future studies should consider longer interventions, incorporate more diverse training approaches, and measure hormonal or molecular markers (e.g., mTOR activity) to clarify the mechanisms underlying the effects of TRE on muscle adaptations.

### 4.3. Hormonal Responses to TRE

The hormones typically associated with metabolism exhibit distinct circadian rhythms, resulting in varying secretion levels at different times of the day. The timing of food intake influences key anabolic and metabolic hormones, including growth hormone (GH), testosterone, and insulin-like growth factor-1 (IGF-1).

GH follows a pulsatile pattern, peaking at night, and is enhanced by prolonged fasting and TRE, aiding fat metabolism and muscle preservation [44]. Testosterone peaks in the morning and declines throughout the day; its availability for muscle repair may be influenced by meal timing [45]. IGF-1 plays a crucial role in muscle hypertrophy and protein synthesis. However, prolonged fasting reduces IGF-1 levels, potentially limiting muscle growth despite TRE’s fat loss benefits [34]. These hormonal responses suggest that eTRE, by aligning food intake with circadian rhythms, may favor fat oxidation, while dTRE, with later feeding times, may disrupt hormonal balance and promote energy storage.

On the other hand, women store fat more efficiently than men due to hormonal and metabolic differences [55]: estrogen enhances fat storage by increasing lipid uptake and reducing lipolysis, especially in the gluteofemoral region [56]. Lower basal testosterone levels in women may lead to reduced anabolic responses to resistance training. These differences may help explain why TRE resulted in weight loss (in the eTRE group) but did not significantly impact the muscle thickness or endurance across groups.

### 4.4. Secondary Findings: Sleep Quality and Adherence

This study also highlights secondary outcomes concerning sleep quality and TRE adherence. The adherence rates were 89.5% and 91.5% for eTRE and dTRE, respectively, corroborating the feasibility and acceptability of TRE in real-life settings. Both TRE groups reported similar durations of sleep, yet it is notable that five participants in the eTRE group reported an improved sleep quality. A similar finding was documented by Kesztyüs, who observed that in a cohort of 99 middle-aged subjects, a three-month TRE intervention (8–9 h) significantly enhanced sleep quality without altering total sleep time [57]. The human circadian system gradually increases melatonin secretion in the evening to facilitate sleep onset and maintenance. However, consuming food late at night may interfere with this circadian rhythm by keeping the digestive system and metabolic pathways more active, which can dampen or delay the normal release of melatonin. By finishing one’s final meal earlier, eTRE potentially aligns the feeding–fasting cycle more closely with endogenous melatonin production, leading to a stronger and more timely rise in melatonin levels. This, in turn, can promote faster sleep onset and deeper sleep.

### 4.5. Limitations and Directions for Future Research

Despite the rigorous study design, several limitations should be acknowledged. First, this study was conducted exclusively on healthy young women to minimize sex-related metabolic variability, which may limit the generalizability of the findings to other populations, such as males, older adults, or individuals with metabolic disorders. Second, the relatively short intervention period (8 weeks) and small sample size (n = 24) may have affected the reliability of the results, particularly in detecting long-term adaptations to resistance training. Third, their dietary intake was not strictly controlled to simulate a real-life TRE implementation better. However, the absence of precise energy intake assessments limits the ability to fully attribute the observed effects to TRE.

Future research should include larger and more diverse samples to confirm the generalizability of these findings. Long-term studies are warranted to assess the sustained effects of TRE on muscle hypertrophy, metabolic outcomes, and training adaptations. Moreover, integrating detailed dietary tracking methods (e.g., 24 h dietary recalls, continuous food diaries) and biomarker assessments (e.g., hormone levels, metabolic rate) will provide deeper insights into the physiological mechanisms underlying TRE-induced adaptations.

## 5. Conclusions

In this eight-week study, early time-restricted eating (eTRE: 8:00 AM–2:00 PM) produced more pronounced weight loss than that with delayed TRE (dTRE: 12:00 PM–6:00 PM) or a control schedule (8:00 AM–8:00 PM) among young women performing resistance training without compromising their muscle thickness or endurance. These findings suggest that aligning meal timing with earlier hours may enhance weight management while preserving muscular adaptations. Further research with larger, more diverse samples and detailed dietary tracking is needed to confirm these results and clarify the underlying hormonal and circadian mechanisms.

## Figures and Tables

**Table 1:** Subjects’ characteristics at baseline.

	eTRE	dTRE	Control
Age (year)	24.1 ± 2.10	23.3 ± 0.89	22.1 ± 2.53
Body Weight (kg)	54.6 ± 2.21	55.7 ± 1.70	55.4 ± 1.79
Height (cm)	163.4 ± 4.74	162.6 ± 3.64	163.2 ± 2.98

Results presented as mean ± SD. Results were not statistically significantly different.

**Table 2:** Changes in body weight, muscle thickness, and push-up performance analyzed using a linear mixed-effects model (LMM).

	eTRE (n = 8)	dTRE (n = 8)	Control (n = 8)	Time	Interaction
Body weight (kg) pre	54.61 ± 2.22	55.68 ± 1.70	55.40 ± 1.79	0.157	0.001 (eTRE)
Body weight (kg) post	52.00 ± 2.96	54.24 ± 1.43	54.92 ± 1.51		0.047 (dTRE)
Muscle thickness (mm) pre	20.61 ± 1.73	20.10 ± 1.25	19.00 ± 1.58	0.001	0.780 (eTRE)
Muscle thickness (mm) post	22.08 ± 1.62	21.46 ± 1.30	20.55 ± 1.49		0.506 (dTRE)
Push-up performance (reps) pre	57.00 ± 16.73	58.75 ± 16.78	64.62 ± 9.40	0.001	0.486 (eTRE)
Push-up performance (reps) post	130.88 ± 38.54	120.62 ± 34.63	132.50 ± 19.23		0.486 (dTRE)

Results are presented as mean ± standard deviation (SD). The “Time” column indicates the main effect of time (pre vs. post), and the “Interaction” column shows the *p*-values for the Time × Group effect in the LMM, with each TRE condition (eTRE and dTRE) compared to the control group. *p* < 0.05 is considered statistically significant.

**Table 3:** Average sleep duration and dietary program adherence.

	eTRE	dTRE	Control
Average sleep duration (h)	7.66 ± 0.52	7.49 ± 0.48	7.58 ± 0.45
Dietary program adherence (%)	85.6	89.5	95.6

Results are not statistically significantly different.

## Data Availability

The dataset supporting this study is openly available in Zenodo at https://doi.org/10.5281/zenodo.14885654.

## Abbreviations

The following abbreviations are used in this manuscript:

TRE	time-restricted eating
eTRE	early time-restricted eating
dTRE	delayed time-restricted eating
SCN	suprachiasmatic nucleus
MyoPS	myofibrillar protein synthesis
RT	resistance training
GH	growth hormone
